# DR^high+^CD45RA^−^-Tregs Potentially Affect the Suppressive Activity of the Total Treg Pool in Renal Transplant Patients

**DOI:** 10.1371/journal.pone.0034208

**Published:** 2012-03-28

**Authors:** Matthias Schaier, Nicole Seissler, Edgar Schmitt, Stefan Meuer, Friederike Hug, Martin Zeier, Andrea Steinborn

**Affiliations:** 1 Department of Nephrology, University of Heidelberg, Heidelberg, Germany; 2 Institute of Immunology, University of Mainz, Mainz, Germany; 3 Institute of Immunology, University of Heidelberg, Heidelberg, Germany; 4 Department of Obstetrics and Gynecology, University of Heidelberg, Heidelberg, Germany; University of California Los Angeles, United States of America

## Abstract

Recent studies show that regulatory T cells (Tregs) play an essential role in tolerance induction after organ transplantation. In order to examine whether there are differences in the composition of the total CD4^+^CD127^low+/−^FoxP3^+^- Treg cell pool between stable transplant patients and patients with biopsy proven rejection (BPR), we compared the percentages and the functional activity of the different Treg cell subsets (DR^high+^CD45RA^−^-Tregs, DR^low+^CD45RA^−^-Tregs, DR^−^CD45RA^−^-Tregs, DR^−^CD45RA^+^-Tregs). All parameters were determined during the three different periods of time after transplantation (0–30 days, 31–1,000 days, >1,000 days). Among 156 transplant patients, 37 patients suffered from BPR. The most prominent differences between rejecting and non-rejecting patients were observed regarding the DR^high+^CD45RA^−^-Treg cell subset. Our data demonstrate that the suppressive activity of the total Treg pool strongly depends on the presence of these Treg cells. Their percentage within the total Treg pool strongly decreased after transplantation and remained relatively low during the first year after transplantation in all patients. Subsequently, the proportion of this Treg subset increased again in patients who accepted the transplant and reached a value of healthy non-transplanted subjects. By contrast, in patients with acute kidney rejection, the DR^high+^CD45RA^−^-Treg subset disappeared excessively, causing a reduction in the suppressive activity of the total Treg pool. Therefore, both the monitoring of its percentage within the total Treg pool and the monitoring of the HLA-DR MFI of the DR^+^CD45RA^−^-Treg subset may be useful tools for the prediction of graft rejection.

## Introduction

Despite the significant improvement in the understanding of allo-immune mechanisms for graft failure and the development of innovative immune-suppressants, graft and patient survival have not increased as expected in the past decade. Prevention of graft rejection and induction of tolerance are common goals in the field of transplantation. Acute rejection has been shown to be one of the strongest negative prognostic factors for long-term graft survival after kidney transplantation [1], [2]. The frequency of acute rejection episodes is highest during the first 6 months after transplantation [3]. During the second and third year post surgery, renal function becomes stable and the incidence of acute rejection and graft loss is markedly reduced [4]. After more than three years, only small changes can be observed in regard to mean GFR decline, annual incidence of graft loss and death, which all were found to represent about 1%. Currently, only limited data exist which could explain this phenomenon. Possibly, several transplant patients can develop tolerance towards the foreign allo-antigens with advancing time after transplantation.

Recent studies show that regulatory T cells (Tregs) play an essential role in tolerance induction after organ transplantation [5], [6]. The majority of such studies were done using animal models. However, in humans, the true function of Tregs in allo-immunity remains in question [7], [8]. Currently, Treg cells are broadly subdivided into those that develop in the thymus (natural (n) Tregs) and those that develop from conventional T-cells in the periphery (induced (i) Tregs) [9]. A specific cell marker that differentiates human nTregs from iTregs is not yet known. Both Treg populations potentially suppress the proliferation of T effector- cells [9] and are characterized by simultaneous expression of the interleukin (IL) 2 receptor α chain (CD25) and the forkhead box P3 (FoxP3) transcription factor [10]. In addition, an inverse correlation between the expression of the IL-7 receptor α chain (CD127) and their suppressive function was observed for CD4^+^CD25^+^ FoxP3^+^-Treg cells [11], [12]. Currently, it is not known, to which extent each of these Treg populations contributes to the prevention of allograft rejection after transplantation. However, there is a growing body of evidence that the suppressive potency of the total Treg cell pool may depend on its composition with distinct Treg subsets. Baecher-Allan et al. have characterized a highly suppressive subset of Treg cells expressing HLA-class II (DR) antigens [13]. Such HLA-DR^+^- Tregs were shown to express higher levels of FoxP3 and induced a more intense and a more rapid T cell suppression than the Tregs that lack HLA-DR expression [13]. Moreover, it is known that the total Treg pool contains a population of naïve CD45RA^+^-Treg cells. Its proportion decreases with increasing age and it was shown that naïve CD45RA^+^-Treg cells were less proliferative than their CD45RO^+^ counterparts [14]. Recent data demonstrate that the suppressive activity of naïve CD45RA^+^-Treg cells is impaired in multiple sclerosis (MS) patients, suggesting that this Treg population may potentially be involved in the pathology of autoimmune diseases [15], [16].

In the present study, we demonstrate that DR^high+^CD45RA^−^-Tregs potentially affect the suppressive activity of the total Treg pool and that the disappearance of this Treg subset gives a strong indication for acute rejection processes.

## Results

### The percentage of CD4^+^CD127^low+/−^FoxP3^+^-Tregs within CD4^+^-T cells is significantly decreased in kidney transplant patients compared to non-transplanted healthy volunteers

**Figure 1:**
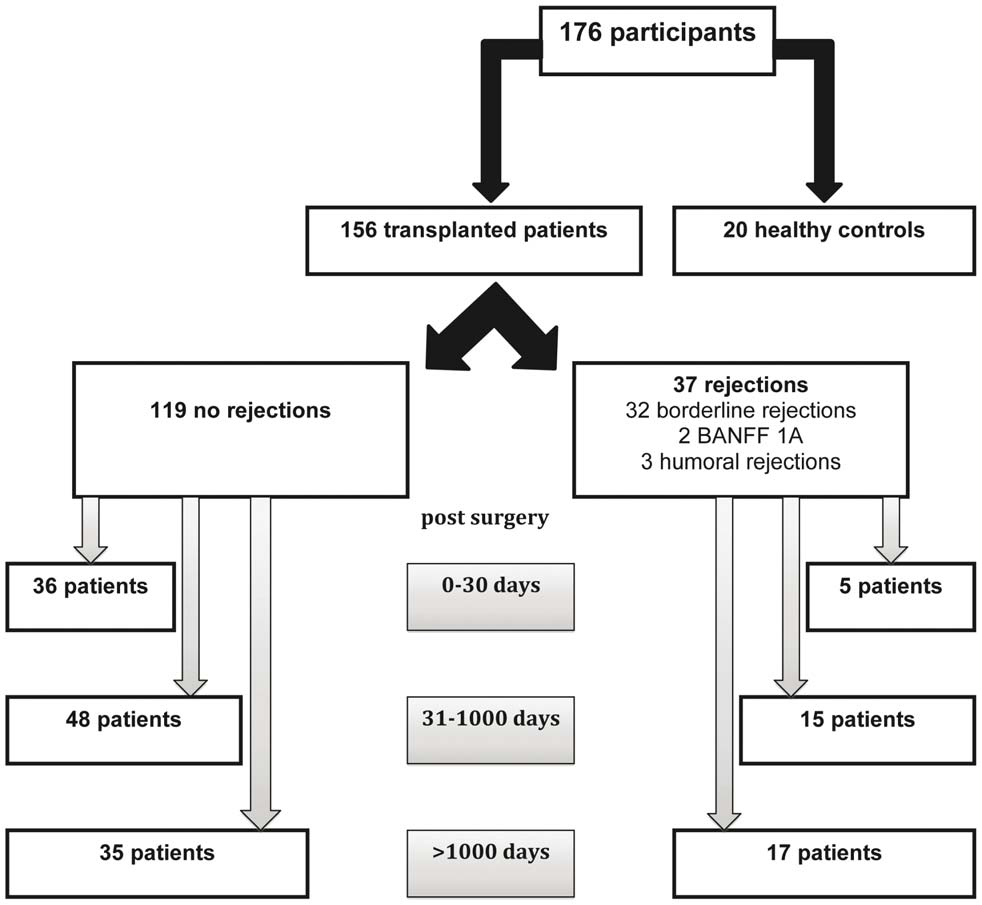
Flow diagram of the different patient groups.

**Figure 2:**
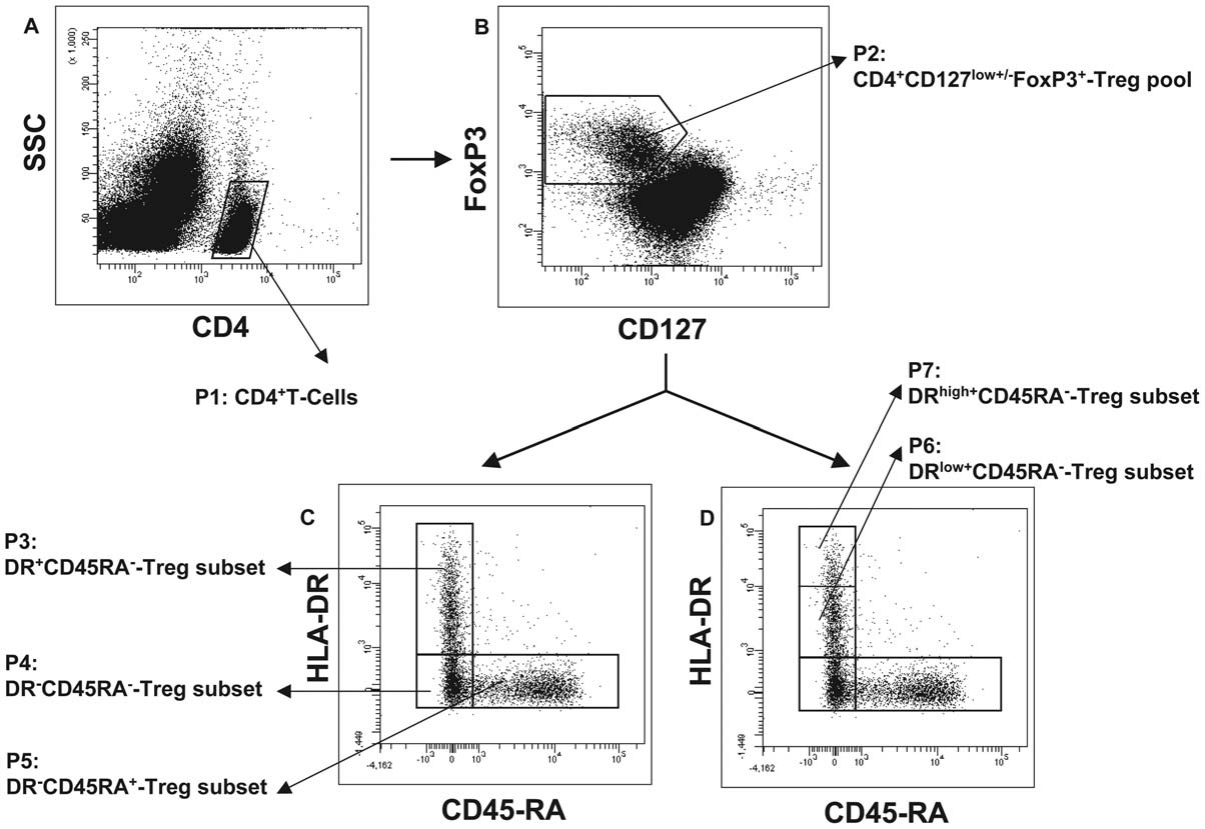
Gating strategy for five color flow cytometric detection of the total CD4^+^CD127^low+/−^FoxP3^+^-Treg cell pool and its composition with four different Treg-subsets. A: CD4^+^-T cells (P1) were gated by fluorescence intensity of CD4 versus side light scatter (SSC). B: CD4^+^CD127^low+/−^FoxP3^+^-Treg cells were gated by fluorescence intensity of FoxP3 versus CD127 (P2). C–D: The percentage of the DR^high+^CD45RA^−^- (P6), the DR^low+^CD45RA^−^- (P7), the DR^−^CD45RA^−^- (P4) and the naïve DR^−^CD45RA^+^- (P5) Treg subset was estimated by analyzing CD4^+^CD127^low+/−^CD25^+^Foxp3^+^-Treg cells (P2) for their expression of HLA-DR and CD45RA. In addition, the MFI of HLA-DR expression of the DR^+^CD45RA^−^FoxP3^+^-Treg subset (P3) was estimated for all participants. MFI = mean fluorescence intensity.

Both the percentage of CD4^+^CD127^low+/−^FoxP3^+^-Treg cells of CD4^+^-T cells and their composition with four distinct Treg cell subsets were determined in the circulation of healthy non-transplanted volunteers (Group A), stable kidney transplant patients (Group B) and kidney transplant patients with biopsy proven rejection (BPR) (Group C), (Table 1, Figure 1). PBMCs obtained from each participant were stained with anti-CD4, anti-CD127, anti-FoxP3, anti-HLA-DR and anti-CD45RA monoclonal antibodies and analyzed by five color flow cytometric analysis. Figure 2 depicts the gating strategy for these measurements. First, PBMCs were analyzed by fluorescence intensity of CD4 versus side light scatter (SSC), (Figure 2A). The CD4^+^-T cells (P1) were gated and analyzed by fluorescence intensity of FoxP3 versus CD127, (Figure 2B). The CD4^+^CD127^low+/−^FoxP3^+^-Tregs were gated (P2) and analyzed by their expression of HLA-DR versus CD45RA (Fig. 2C). By that, three distinct Treg cell subsets became apparent: DR^+^CD45RA^−^-Tregs (P3), DR^−^CD45RA^−^-Tregs (P4) and naïve DR^−^CD45RA^+^-Tregs (P5). The percentages of these distinct Treg subsets within the total CD4^+^CD127^low+/−^FoxP3^+^-Treg cell pool were estimated for all participants. In addition, the level of HLA-DR expression (HLA-DR MFI) of the DR^+^CD45RA^−^-Treg subset (Fig. 2C, P3) and the percentages of DR^low+^CD45RA^−^-Tregs (Fig. 2D, P6) and DR^high+^CD45RA^−^-Tregs (Fig. 2D, P7) of the total Treg cell pool (P2) were documented.

**Table 1:** Clinical characteristics of kidney transplant patients.

Group	n	Median time after Transplantation [days]	Median Creatinine [mg/dl]	Results of Biopsy	Immunosuppression
A	20	-	-	-	-
B	119	181 [5–9155]	1.84 [0.74–11.6]	**no rejection**	
					27 Tac+MPA+steroids
					80 CsA+MPA+steroids
					5 mTor+MPA+steroids
					4 mTor+CsA+MPA+steroids
					3 others+MPA+steroids
C	37	793 [6–6938]	2.41 [1.34–6.93]	**acute rejection**	
				32 borderline rejections	
					8 Tac+MPA+steroids
					22 CsA+MPA+steroids
					1 mTor+CsA+MPA+steroids
					1 Azathioprin+MPA+steroids
				2 BANFF 1A rejections	
					1 CsA+MPA+steroids
					1 mTor+CsA+MPA+steroids
				3 acute humoral rejections	
					1 CsA+MPA+steroids
					1 mTor+MPA+steroids
					1 mTor+CsA+MPA+steroids

**Figure 3:**
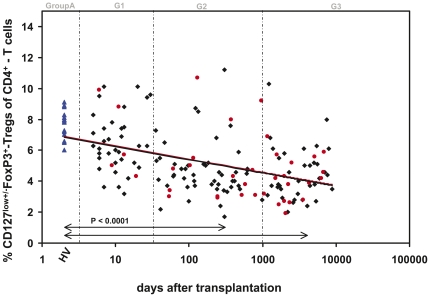
Detection of the percentage of CD4^+^CD127^low+/−^FoxP3^+^-Treg cells of total CD4^+^-T cells in rejecting and non-rejecting patients after kidney transplantation. The percentage of CD4^+^CD127^low+/−^FoxP3^+^-Tregs of total CD4^+^-T cells was estimated in healthy non-transplanted volunteers (▴), in kidney transplant patients with stable transplant function (♦) and in kidney transplant patients with biopsy proven rejection (BPR), (•). The percentage of CD4^+^CD127^low+/−^FoxP3^+^-Tregs decreased continuously after transplantation. Significant differences between rejecting and non-rejecting patients were not detected.

Figure 3 shows the percentages of CD4^+^CD127^low+/−^FoxP3^+^-Treg cells within the total CD4^+^-T cell pool in healthy non-transplanted volunteers (Group A), in stable kidney transplant patients (Group B) and in transplant patients with biopsy proven rejection (BPR) (Group C). Compared to healthy non-transplanted volunteers, the percentage of CD4^+^CD127^low+/−^FoxP3^+^-Tregs decreased continuously after transplantation. Significant differences concerning the percentage of CD4^+^CD127^low+/−^FoxP3^+^-Tregs at different time points (G1–G3) after transplantation between rejecting and non-rejecting patients were not detected.

### The suppressive activity of the CD4^+^CD127^low+/−^CD25^+^-Treg cell pool is significantly reduced in patients with biopsy proven rejection (BPR)

**Figure 4:**
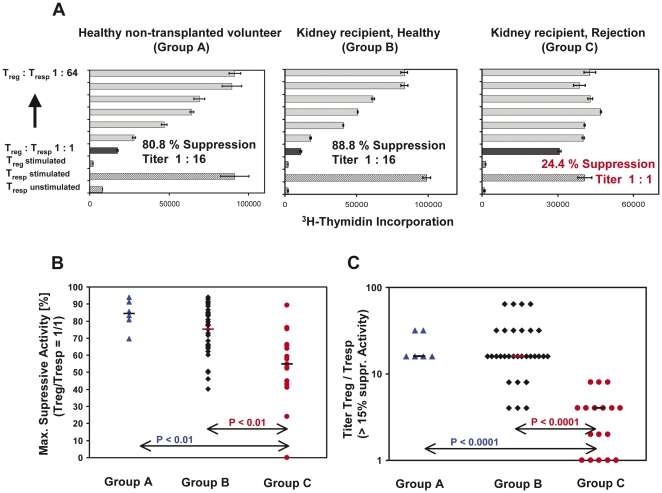
Evaluation of the suppressive activity of CD4^+^CD127^low+/−^CD25^+^-Treg cells obtained from healthy non-transplanted volunteers and kidney transplant patients with stable transplant function or acute rejection. A: CD4^+^CD127^low+/−^CD25^+^-Treg cells were isolated by the MACS technique and their suppressive activity was examined using suppression assays (see methods). One representative experiment is shown for healthy volunteers, kidney recipients with stable transplant function and kidney recipients with acute rejection. The maximum suppressive activity (Treg/Tresp = 1/1) (B) and the ratio of Treg/Tresp (titer) up to which the purified Treg cells could be diluted to achieve a minimum suppressive activity of at least 15% (C), were estimated for all participants. The figures show the individual and median data obtained for all patients groups.

To examine whether there were differences in the suppressive activity of the total Treg pool between rejecting and non-rejecting transplant patients we used coculture suppression assays described in the methods section. To evaluate the suppressive capacity of CD4^+^CD127^low+/−^CD25^+^-Tregs, obtained from the different patient groups (Group A–C), we determined the maximum suppressive activity (ratio of Treg cells to responder T (Tresp) cells 1∶1) and calculated the ratio of Treg cells to Tresp cells that resulted in a suppression of at least 15%. Figure 4A depicts the results of one representative experiment obtained for healthy non-transplanted volunteers (Group A), stable kidney transplant patients (Group B) and transplant patients with BPR (Group C), respectively. Figure 4B and 4C summarize the data for the individual participants in each of these three patient groups. We found that the suppressive activity of the isolated CD4^+^CD127^low+/−^CD25^+^-Tregs, obtained from stable transplant recipients, was in the same range as that of CD4^+^CD127^low+/−^CD25^+^-Tregs obtained from healthy non-transplanted volunteers. In contrast, the suppressive activity of Tregs obtained from patients with acute rejection was significantly reduced compared to healthy non-transplanted controls and to stable transplant patients, (Figure 4B). Furthermore, the ratio of Treg cells to responder cells (Titer Treg/Tresp) leading to a suppression of at least 15%, was significantly decreased in patients with acute rejection compared to healthy controls and stable transplant patients (Figure 4C).

### After transplantation, the composition of the total Treg cell pool changes characteristically

**Figure 5:**
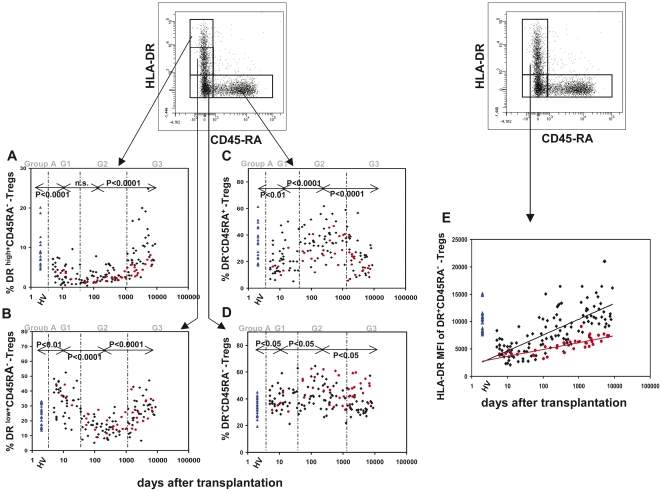
Detection of the changes in the composition of the total CD4^+^CD127^low+/−^FoxP3^+^-Treg cell pool with four different Treg subsets during the time after transplantation. The percentage of the DR^high+^CD45RA^−^- (A), the DR^low+^CD45RA^−^- (B), the DR^−^CD45RA^+^- (C), and the DR^−^CD45RA^−^- (D) Treg subset within the total Treg cell pool was estimated in healthy non-transplanted volunteers (▴) and in non-rejecting (♦) and rejecting kidney transplant patients (•) at different time points after transplantation. In addition the HLA-DR MFI of the DR^+^CD45RA^−^-Treg subset was determined for all participants in all patient groups (E). Monitoring the HLA-DR MFI allowed a significant discrimination between rejecting and non-rejecting patients, due to a significantly reduced percentage of the DR^high+^CD45RA^−^-Treg subset within the total Treg pool. MFI = mean fluorescence intensity.

In order to examine whether there were changes in the composition of the total Treg cell pool with distinct Treg subsets (DR^high+^CD45RA^−^-Tregs, DR^low+^CD45RA^−^-Tregs, DR^−^CD45RA^−^-Tregs and naïve DR^−^CD45RA^+^-Tregs), we determined their percentages in healthy controls and stable transplant patients. Figures 5A-4D summarize the results of these analyses. The individual ratios of the four Treg subsets showed characteristic changes over time in transplanted patients. Therefore, the transplant patients were grouped depending on the period of time after surgery (G1: 0–30 days, G2: 31–1000 days, G3: >1000 days). Non-transplanted volunteers served as healthy controls (Group A). Compared to healthy non-transplanted controls (Group A), the percentage of DR^high+^CD45RA^−^-Tregs decreased strongly within the first 30 days after transplantation (Fig. 5A, G1) and remained at minimum levels up to 1000 days post surgery (Fig. 5A, (G2)). The percentage of DR^low+^CD45RA^−^-Tregs increased initially (Fig. 5B, G1), but decreased subsequently and reached the lowest levels between 31 and 1000 days post transplantation (Fig. 5B, (G2)). After a period of 1000 days (G3) both the percentages of DR^high+^CD45RA^−^-Tregs and DR^low+^CD45RA^−^-Tregs returned to levels in the range of healthy non-transplanted controls (Group A), (Figures 5A and 5B). As the differentiation between DR^high+^CD45RA^−^-Tregs and DR^low+^CD45RA^−^-Tregs was difficult, we additionally estimated the HLA-DR mean fluorescence intensity (MFI) of the DR^+^CD45RA^−^-Treg cell subset for all participants (Fig. 5E). We found a dramatic decrease within the first days after surgery (Fig. 5E, G1), it is likely due to a strong decrease of the DR^high+^CD45RA^−^-Treg subset and an increase of the DR^low+^CD45RA^−^-Treg subset. However, subsequently a continuous increase of the HLA-DR MFI could be documented (Fig. 5E, G2–G3), because in the period between 30–1000 days post surgery (Fig. 4E, G2), the percentage of the DR^low+^CD45RA^−^-Treg subset decreased while the percentage of DR^high+^CD45RA^−^-Treg subset did not change. After 1000 days post surgery (Fig. 5E, G3), both the percentages of the DR^high+^CD45RA^−^- and the DR^low+^CD45RA^−^-Treg subsets increased strongly.

The percentage of the naïve DR^−^CD45RA^+^ -Treg subset decreased significantly during the first 30 days after transplantation (Fig. 5C, G1), but afterwards, it increased strongly and reached maximum levels between 31 and 1000 days post surgery (Fig. 5C, G2). After 1000 days post surgery, a significant decrease of this Treg subset was observed (Fig. 5C, G3).

The percentage of the DR^−^CD45RA^−^-Treg subset increased immediately after transplantation (Fig. 5D, G1) and revealed the highest levels between 31 and 1000 days after surgery (Fig. 5D, G2). After more than 1000 days post transplantation (Fig. 5D, G3), a slight but significant decrease was observed.

### The percentage of the DR^high+^CD45RA^−^-Treg subset within the total Treg pool and the HLA-DR MFI of the DR^+^CD45RA^−^-Treg subset are strongly reduced in patients with BPR after 31 days post surgery

In order to examine whether there are differences in the composition of the total Treg cell pool between stable transplant patients (Group B) and patients with BPR (Group C), we compared the percentages of the different Treg cell subsets (DR^high+^CD45RA^−^-Tregs, DR^low+^CD45RA^−^-Tregs, DR^−^CD45RA^−^-Tregs, DR^−^CD45RA^+^-Tregs) and the HLA-DR MFI of the DR^+^CD45RA^−^-Treg cell subset. All parameters were determined during the three different periods of time after transplantation (G1: 0–30 days, G2: 31–1000 days, G3: >1000 days). Figure 5A–5E and Table 2 show the results of these measurements.

**Table 2:** Percentage of different Treg subsets of the total Treg pool obtained from healthy non-transplanted volunteers and kidney transplant patients in the presence or absence of BPR.

	Time after Tx [days]	n	DR^−^CD45RA^+^[% Tregs]	DR^−^CD45RA^−^[% Tregs]	DR^low+^ CD45RA^−^[% Tregs]	DR^high+^ CD45RA^−^[% Tregs]	HLA-DR MFI
**Controls**		20	34 (17–61)	33 (19–45)	25 (14–33)	7.5 (4.5–20.1)	10470 (7607–15090)
**No BPR**	0–30	36	20 (5–50)	37 (21–59)	34 (14–53)	2.8 (0.5–9.6)	4604 (2112–9076)
	31–1000	48	35 (13–62)	42 (27–65)	16 (5–29)	2.5 (0.4–7.8)	7431 (2782–16440)
	>1000	35	24 (7–59)	33 (22–57)	28 (7–47)	8.4 (2.1–24.8)	11102 (7219–21025)
**BPR**	0–30	5	16 (10–30)	35 (30–45)	37 (30–47)	3.6 (2.0–4.3)	4058 (4002–4817)
	31–1000	15	28 (12–47)*	51 (34–62)*	16 (9–27)	1.7 (1.0–2.8)*	5151 (3230–6401)*
	>1000	17	21 (6–41)	46 (32–60)***	26 (14–41)	4.7 (2.2–8.1)***	6843 (5312–9105)***

* : p<0.05; no BPR versus BPR.

*** : p<0.001; no BPR versus BPR.

Among 156 transplant patients, 37 patients suffered from BPR. The most prominent differences between non-rejecting (Group B) and rejecting BPR patients (Group C) were seen regarding the DR^high+^CD45RA^−^-Treg cell subset. BPR patients showed a 32% reduction of this Treg subset within the total Treg cell pool after 31–1000 days (G2) and a 44% reduction after more than 1000 days post surgery (G3) versus patients without rejection. Differences concerning the DR^low+^CD45RA^−^-Treg subset could not be detected. In parallel, the HLA-DR MFI of the DR^+^CD45RA^−^-Treg cell subset was strongly reduced in patients with BPR versus no BPR (31% after 31–1000 days (G2); 38% after 1000 days (G3)). Figure 4E shows the relationship of the HLA-DR MFI of the DR^+^CD45RA^−^-Treg cell subset and the time after transplantation for stable transplant patients (Group B) and for patients with BPR (Group C). A positive linear correlation was found for stable transplant patients (r = 0.569, p<0.00001) and for patients with BPR (r = 0.664, p<0.00001). Comparison of these two regression lines revealed significant differences (p<0.001) between rejecting and non-rejecting transplant patients.

Moreover, contrary to stable transplant patients, BPR patients revealed a significantly higher proportion of DR^−^CD45RA^−^-Tregs after 30 days post surgery. Their percentage within the total Treg cell pool was 21% higher after 31 days (G2) and 39% higher after 1000 days post transplantation (G3). The percentage of the naïve DR^−^CD45RA^+^-Treg subset was lower in patients with BPR after 30 days post surgery, compared to patients with stable transplant function (G2 and G3). With regard to all examined parameters, there were no differences between rejecting and non-rejecting transplant patients during the first 30 days after surgery (G1).

### The low suppressive capacity of CD4^+^CD127^low+/−^CD25^+^-Tregs observed in BPR patients correlates with a decline of the HLA-DR MFI of the DR^+^CD45RA^−^-Treg subset

**Figure 6:**
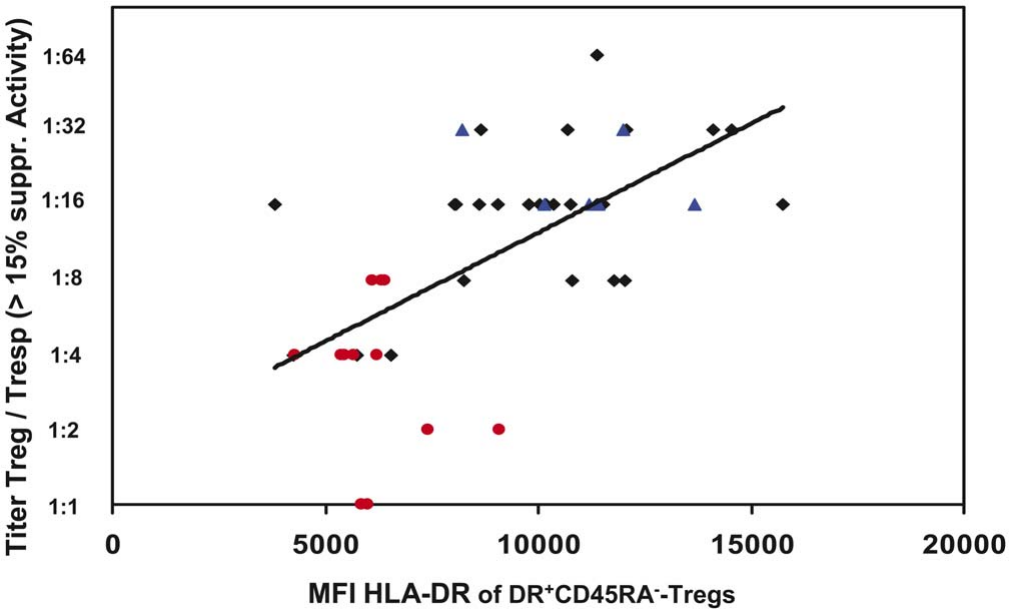
Correlation between the HLA-DR MFI of the DR^+^CD45RA^−^-Treg subset and the ratio of Treg/Tresp (titer) up to which a significant suppression could be achieved. CD4^+^CD127^low+/−^CD25^+^-Tregs were purified from healthy non-transplanted volunteers (▴), kidney transplant patients with stable transplant function (♦) and kidney transplant patients with BPR (•). Their suppressive activity concerning the ratio of Treg/Tresp (titer) up to which the purified Treg cells could be diluted to achieve a minimum suppressive activity of at least 15% was related to the HLA-DR MFI of the DR^+^CD45RA^−^-Treg subset. The figure shows the positive correlation (r = 0.546, p<0.001) between the HLA-DR MFI of the DR^+^CD45RA^−^-Treg subset and the ratio of Treg/Tresp (titer). MFI = mean fluorescence intensity.

We examined whether there was a correlation between the HLA-DR MFI of the DR^+^CD45RA^−^-Treg subset and the suppressive activity of the total Treg cell pool. Figure 6 shows the positive correlation (r = 0.546, p<0.001) between the HLA-DR MFI of the DR^+^CD45RA^−^-Treg subset and the titer (Treg/Tresp) of Tregs, with which a minimum suppressive activity of 15% was achieved. Thereby, CD4^+^CD127^low+/−^CD25^+^-Tregs were obtained from healthy controls, BPR patients and patients without BPR. In summary, our data clearly demonstrate that the low suppressive activity of CD4^+^CD127^low+/−^CD25^+^-Tregs observed in BPR patients correlates with a low HLA-DR MFI of the DR^+^CD45RA^−^-Treg cells subset. Otherwise, the high suppressive activity assessed in healthy controls and stable transplant patients correlate with a high HLA-DR MFI of the DR^+^CD45RA^−^-Treg subset.

### The DR^high+^CD45RA^−^-Treg subset has the highest suppressive capacity within the total CD4^+^CD127^low+^CD25^+^-Treg cell pool

**Figure 7:**
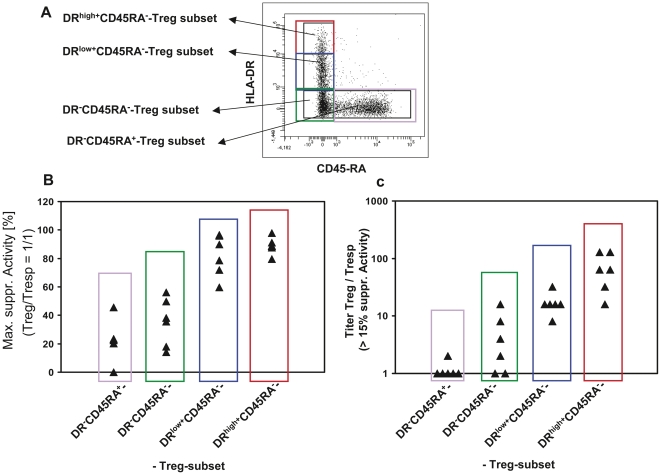
Positive selection and functional testing of four different Treg subsets within the total CD4^+^CD127^low+/−^CD25^+^-Treg pool. A: Magnetically isolated CD4^+^CD127^low+/−^CD25^+^-Treg cells were stained with anti-HLA-DR and anti-CD45RA specific monoclonal antibodies and sorted into a population of DR^high+^CD45RA^−^-, DR^low+^CD45RA^−^-, DR^−^CD45RA^−^-, and naïve DR^−^CD45RA^+^-Treg cells. Subsequently the different Treg populations obtained from six different healthy non-transplanted volunteers were analyzed concerning their maximum suppressive activity (Treg/Tresp = 1/1) (B) and the minimum ratio of Treg/Tresp (titer) up to which the purified Tregs could be diluted to achieve a minimum suppressive activity of at least 15% (C).

Our data demonstrate that the level of HLA-DR expression of the DR^+^CD45RA^−^-Treg cell subset correlated positively with the suppressive activity of the total Treg cell pool from healthy controls, stable transplant patients and patients with BPR, respectively. These findings suggested that the DR^high+^CD45RA^−^-Treg subset has the highest suppressive activity within the total Treg cell pool. Thus, magnetically isolated CD4^+^CD127^low+/−^CD25^+^-Treg cells were separated via FACSort into four different Treg cell subsets consisting of DR^high+^CD45RA^−^-, DR^low+^CD45RA^−^-, DR^−^CD45RA^−^- and naïve DR^−^CD45RA^+^-Treg cells (Figure 7A). Subsequently, the suppressive activity of these different Treg subsets was analyzed. Both the maximum suppressive activity (Figure 7B) and the titer (Treg/Tresp) with which a minimum suppressive activity of 15% could be achieved (Figure 7C) were highest for the DR^high+^CD45RA^−^-Treg subset. For the DR^low+^CD45RA^−^-Treg subset, the suppressive activity was slightly reduced compared to the DR^high+^CD45RA^−^-Treg subset. An even less suppressive activity was found for the DR^−^CD45RA^−^-Tregs and the lowest suppressive activity was assessed for the naïve DR^−^CD45RA^−^-Treg subset. Thus, the DR^high+^CD45RA^−^-Treg subset was shown to exhibit the highest suppressive activity of the total CD4^+^CD127^low+/−^CD25^+^-Treg cell pool.

## Discussion

Therefore, Tregs play an important role in transplantation [5], [17] and pregnancy [18], [19], but also influence infectious diseases [20], autoimmunity [21], and anti-tumor immunity [22]. Meanwhile, promising data in regard to solid organ transplantation are increasingly available. Considering the potential role of Tregs in the control of allo-responses in renal transplant patients, one of the aims of this study was to examine whether quantitative and functional monitoring of Tregs could be used as markers of immunological tolerance. We demonstrated that in post-transplant patients the proportion of CD4^+^CD127^low+/−^FoxP3^+^-Tregs decreased continuously in comparison to healthy volunteers over an observation period of almost 25 years after transplantation. In contrast to the results obtained with smaller studies [23], [24], [25], we demonstrated in our cohort that the percentage of CD4^+^CD127^low+/−^FoxP3^+^-Tregs within the CD4^+^-T cells was not different between rejecting and non-rejecting patients. Such differences may be based on inconsistent characterization of the total Treg pool, different gating strategies and small numbers of participants in many studies.

In addition, we demonstrated that the suppressive activity of the total CD4^+^CD127^low+/−^CD25^+^-Treg cell pool from patients with biopsy proven rejection (BPR) was significantly reduced compared to patients with stable graft function and compared to healthy non-transplanted volunteers. Currently, limited data exist concerning the suppressive activity of Tregs from transplant patients with BPR. Dijke et al. demonstrated that CD4^+^CD25^+^FoxP3^+^ Tregs of heart transplant patients who experienced acute rejection had a reduced regulatory function compared to those obtained from non-rejecting patients [26]. These and our results are contrary to the findings of a smaller study performed by Kreijveld et al. who did not find any differences concerning the suppressive activity between renal transplant recipients with rejection and those without rejection [27].

Moreover, recent data revealed that the total Treg pool seems to be inconsistent. Different Treg subsets such as naïve CD45RA^+^- or HLA-DR^+^- expressing Treg cells were shown to be important for the functional activity of the total Treg cell pool [13], [15]. We demonstrated that differential expression of HLA-DR and CD45RA distinguished four different Treg subsets, which showed characteristic changes concerning their percentages within the total Treg pool during the time after transplantation. Initially after transplantation, the proportion of the naïve DR^−^CD45RA^+^- and the DR^high+^CD45RA^−^-Tregs decreased strongly while the proportion of the DR^low+^CD45RA^−^- and the DR^−^CD45RA^−^-Tregs showed a considerable increase. In consequence, the HLA-DR MFI of the DR^+^CD45RA^−^-Treg subset was also dramatically reduced. Such findings may propose that there is a strong conversion of naïve Tregs into DR^−^CD45RA^−^- and DR^low+^CD45RA^−^-Tregs shortly after transplantation. In addition, we observed the striking effect that the naïve DR^−^CD45RA^+^-Treg subset increased strongly during the first year but decreased considerably during the time afterwards. Complementary to the naïve DR^−^CD45RA^+^-Treg subset the DR^low+^CD45RA^−^-Treg subset decreased significantly during the first year but increased during the time afterwards. Such findings may propose that naïve DR^−^CD45RA^+^-Treg cells may be increasingly released by the thymus during the first year after transplantation. Presumably, after that time, the thymic output of Treg cells is exhausted. As HLA-DR^+^-Treg cells are known to represent highly activated mature Treg cells [13], it seems likely that during the first year after transplantation such cells were preferentially eliminated, presumably due to permanent allogenic stimulation of the immune system. After that time, the maturation of the HLA-DR^+^-Treg cells outnumbers their elimination and the percentage of the HLA-DR^+^-Treg cells again increases to the level of healthy non-transplanted volunteers. In either case it seems that the changes in the percentages of the DR^high+^- and DR^low+^CD45RA^−^-Treg cells occur in the way that the HLA-DR MFI increases continuously after transplantation. Such findings may explain why transplant outcome studies have shown markedly reduced rejection rates after three years post surgery [3], [4].

To determine whether there were differences in the composition of the total Treg cell pool between transplant patients with and without BPR, we determined the proportion of the different Treg cell subsets for both patient collectives. Comparable to previous works [27], we found a reduction of the naïve DR^−^CD45RA^+^-Treg subset in regard to the total Treg pool in patients with BPR compared to patients with stable transplant function. Rejecting patients showed significantly higher proportions of DR^−^CD45RA^−^-Treg cells, but the most prominent difference between rejecting and non-rejecting patients was seen regarding the HLA-DR MFI of the DR^+^CD45RA^−^-Treg cell subset. This effect may be explained by the fact that patients with allograft rejection showed a highly significant decrease of the DR^high+^CD45RA^−^-Treg subset within the total Treg cell pool.

The evaluation of the suppressive activity of each of the four different Treg subsets (DR^high+^CD45RA^−^-, DR^low+^CD45RA^−^-, DR^−^CD45RA^−^- and DR^−^CD45RA^+^-Tregs) revealed that the DR^high+^CD45RA^−^-Treg subset had the highest suppressive activity compared to the other Treg subsets.

Similar to findings documented by Baecher-Allan [13], the populations of DR^high+^CD45RA^−^-Tregs and DR^low+^CD45RA^−^-Tregs had a higher suppressive capacity than the populations of DR^−^CD45RA^−^- and DR^−^CD45RA^+^-Tregs. Therefore, specifically the loss of DR^high^ CD45RA^−^-Tregs obviously impaired the suppressive activity of the total Treg cell pool. Our data clearly demonstrate that the determination of the HLA-DR MFI allows a precise evaluation of the ratio between DR^high+^CD45RA^−^-Tregs and DR^low+^CD45RA^−^-Tregs within the DR^+^CD45RA^−^-Treg cell subset. We found a significant correlation between the level of HLA-DR expression of the DR^+^CD45RA^−^-Treg subset and the suppressive activity of the total Treg pool, determined both in healthy non-transplanted volunteers, healthy transplanted patients and transplanted patients with BPR. The lowest suppressive activity of the CD4^+^CD127^low+/−^CD25^+^-Treg pool which correlated with a low HLA-DR MFI of the DR^+^CD45RA^−^-Treg cell subset was observed in patients with BPR.

During pregnancy the “fetal semi-allograft” is perfectly tolerated by the maternal immune system. In a recent published study we demonstrated that women with preterm labor necessitating preterm delivery showed significantly reduced HLA-DR expression of CD4^+^CD127^low+/−^CD25^+^FoxP3^+^-Tregs, indicating that the immunologic mechanisms leading to preterm labor may be similar to those leading to allograft rejection after transplantation [28]. The underrepresentation of HLA-DR^+^-Treg cells in the neonatal compared with the adult circulation indicates that the presumed nTreg population gains HLA-DR expression upon differentiation [29]. Ashley et al. suggest that the CD127^low^DR^+^-Tregs are terminally differentiated effector Tregs, as they do not proliferate and are highly sensitive to apoptosis [30]. It was shown that Granzyme B, which was produced by strongly stimulated non-regulatory responder CD4^+^-T cells, reduced especially the suppressive capacity of the non-proliferating HLA-DR^+^-Tregs cells [30]. On the other hand, the proliferation-competent HLA-DR^−^-Treg cells remained viable [30] and were shown to be more sensitive to Fas-L induced apoptosis [31]. Therefore, it may be hypothesized that the decrease in the MFI of the DR^+^CD45RA^−^-Treg cell subset obtained from rejecting kidney recipients may be caused by the increased Granzyme B induced apoptosis of DR^+^CD45RA^−^-Treg cells which express the HLA-DR molecules very strongly. As it was ascertained that the Granzyme B induced apoptosis of the highly suppressive HLA-DR^+^-Treg cells happened preferentially in the case of strong responder T-cell stimulation [30], it may be assumed that acute rejection episodes after transplantation that are characterized by strong stimulation of CD4^+^-T cells lead to a loss of the strongly suppressive DR^high+^-Treg-subset.

In summary, we clearly demonstrated that patients with biopsy proven rejection (BPR) show deficiencies concerning the functional activity of their Treg pool. Thereby, its composition was changed in the way that the DR^high+^CD45RA^−^-Treg subset, which was shown to possess the highest suppressive activity, was decreased. In contrast, the DR^−^CD45RA^−^-Treg subset with lower suppressive capacity was increased in transplanted patients with acute rejection. Especially the determination of the HLA-DR MFI of the DR^+^CD45RA^−^-Treg subset allowed a significant discrimination between patients with acute graft rejection and those without rejection. The clinical usefulness of the monitoring of these peripheral blood parameters after solid organ transplantation and its relation to clinical outcomes needs to be investigated in large prospective cohort studies of transplant patients.

## Materials and Methods

### Study population

The study was approved by the Ethics Committee of the Medical Faculty Heidelberg. All patients and healthy controls were fully informed of the aim of the study and written informed consent was obtained from all participants.

The study included 20 non-transplanted healthy volunteers (Group A) and 156 kidney transplant patients (Groups B and C) (Table 1). Forty-seven patients received a graft obtained from a living donor and 109 patients received a graft from a cadaveric donor. The mean age of our transplant cohort was 45 years (20–73 years). The causes of end-stage renal failure in our transplant cohort were diabetes mellitus (38%, n = 59), vascular nephopathy (26%, n = 41), glomerulonephritis (15%, n = 23), autosomal dominant polycystic kidney disease (8%, n = 12), autoimmune diseases (5%, n = 8) like systemic lupus erythematosus and systemic vasculitis and 8% (n = 13) were unknown. Blood samples were obtained on the same day when kidney transplant recipients admitted to the Department of Nephrology for kidney biopsy. Kidney biopsies were classified according to the BANFF-classification [32], [33]. Biopsy proven rejection was defined as rising creatinine over 30% above the last three measurements and further pathological findings according to the BANFF-classification (Table 1). Patients with acute graft failure because of infection, postrenal obstruction or drug induced renal failure were excluded. All transplant patients were further subdivided into three different groups. The first group (G1) contains transplant recipients before the 30th day after transplantation. The second group (G2) contains recipients between the 31st and the 1000th day after transplantation and the third group (G3) contains recipients more than 1000 days post transplantation. The 156 transplant patients received an immunosuppressive regime with Mycophenolic acid (MPA), Methylprednisolone and Calcineurininhibitors (Ciclosporin: 104 patients; Tacrolimus: 35 patients) or mTOR-Inhibitors (Everolimus: 13 patients, whereas 7 recipients received a combination of Cyclosporine and Everolimus) and 4 recipients received other immunosuppressive drugs (Table 1).

### Fluorescence-activated cell sorter (FACS) staining

Venous blood samples (9 ml) from all patients were collected into EDTA-containing tubes. Whole peripheral blood mononuclear cells (PBMCs) were isolated by Ficoll-Hypaque (Amersham Bioscience) gradient centrifugation and analyzed by five color flow cytometric analysis. Briefly, PBMCs (4×10^6^ cells) were surface-stained with 10 µl PerCP-conjugated-anti-CD4 (BD Bioscience), 10 µl PE-conjugated anti-CD127 (eBioscience), 5 µl PE-Cy7-conjugated anti-HLA-DR (BD Bioscience) and 20 µl APC-conjugated anti-CD45RA (BD Bioscience) mouse monoclonal antibodies. Intracellular staining for the detection of FoxP3 was achieved using a FITC labeled anti-human FoxP3 staining set (clone PCH101, eBioscience) according to the manufacturer's instructions. Both the percentage of CD4^+^CD127^low+/−^FoxP3^+^-Treg cells of total CD4^+^-T cells and the percentage of DR^high+^CD45RA^−^-Tregs, DR^low+^CD45RA^−^-Tregs, DR^−^CD45RA^−^-Tregs and naïve DR^−^CD45RA^+^-Tregs within the total Treg pool were estimated for all participants. In addition the mean fluorescence intensity (MFI) of HLA-DR expression of the DR^+^CD45RA^−^-Treg cell subset was estimated for all participants. Negative control samples were incubated with isotype-matched antibodies. Dead cells were excluded by forward and side scatter characteristics. Cells were analyzed by a FACS Canto cytometer (BD Bioscience). Cells were analyzed by a FACS Canto cytometer (BD Bioscience) which is equipped with a 488-nm blue laser and a 633-nm red laser. The following standard filter set-ups were used: PerCP: 655 Longpass/670 LP Bandpass; PE: 556 Longpass/585/42 Bandpass; FITC: 502 Longpass/530/30 Bandpass; PE-Cy7: 735 Longpass/780/60 Bandpass; APC: 660/20 Bandpass). Statistical analysis was based on at least 100,000 gated CD4^+^-T cells.

### Positive selection and staining of CD4^+^CD127^low+/−^CD25^+^-Treg cells

Whole peripheral blood mononuclear cells (PBMCs) were isolated from 45 ml EDTA-blood samples by Ficoll-Hypaque (Amersham Bioscience) gradient centrifugation. The CD4^+^CD127^low+/−^CD25^+^-Treg cells were purified using the CD4^+^CD127^low+/−^CD25^+^-Regulatory T cell Isolation Kit II (Miltenyi Biotec) according to the manufacturer's instructions. First CD4^+^CD127^low+/−^-T-cells were isolated by magnetic depletion of non-CD4^+^CD127^high+^-T-cells. In a second step, the CD4^+^CD127^low+/−^CD25^+^-Treg cells were isolated by positive selection over two consecutive columns. The CD4^+^CD127^low+/−^CD25^−^-T cells were obtained in the flow-through fraction and used as responder T cells. The CD4^+^CD127^low+/−^CD25^+^-Treg cells were subsequently retrieved from the columns. The purified CD4^+^CD127^low+/−^CD25^+^-Treg cell fraction was analyzed using four color flow cytometry. Briefly, 1×10^5^ cells were stained with 10 µl PerCP-conjugated-anti-CD4, PE-conjugated anti-CD25, FITC-conjugated FoxP3 and biotin-conjugated anti-CD127 monoclonal antibodies. Positive staining for CD127 was detected using APC-conjugated streptavidin molecules. On average, 85% of the isolated CD4^+^CD127^low+/−^CD25^+^-Treg cells were shown to be within the CD4^+^CD127^low+/−^CD25^+^Foxp3^+^-Treg cell population.

### Co-culture suppression assay

Whole peripheral mononuclear cells (PBMCs) were isolated from 45 ml peripheral blood drawn in EDTA tubes by Ficoll-Hypaque (Amersham Bioscience) gradient centrifugation. CD4^+^CD127^low+/−^CD25^+^-Treg cells were purified using the CD4^+^CD127^low+/−^CD25^+^-Regulatory T cell Isolation Kit II (Miltenyi Biotec) described above. In all assays, 2×10^4^ responder-T-cells were co-cultured with the purified CD4^+^CD127^low+/−^CD25^+^-Treg cells at ratios 1∶1 to 1∶256 in 96-well v-bottom plates. Suppression assays were performed in a final volume of 100 µl/well of X-VIVO15 medium (Bio Whittaker). For T-cell stimulation, the medium was supplemented with 1 µg/ml anti-CD3 and 2 µg/ml anti-CD28 antibodies (eBioscience). As controls, CD4^+^CD127^low+/−^CD25^+^-Treg cells and responder T cells alone were cultured both with and without any stimulus. Cells were incubated at 37°C and 5% of CO_2_. After four days, 1 µCi ^3^H-thymidine was added to the cultures and cells were further incubated for 16 hours. Then, cells were harvested and ^3^H incorporation was measured by scintillation counting. All assays exhibited <10% SEM and were performed a minimum of six times using blood from 6 different healthy non-transplanted volunteers, 32 healthy non-rejecting transplant patients and 17 transplant patients with acute rejection. In order to compare the suppressive capacity of the isolated CD4^+^CD127^low+/−^CD25^+^-Tregs between the different patient groups, we calculated the maximum suppressive activity (ratio of Treg cells to responder T cells 1∶1) and the minimum ratio of Treg cells to responder cells, with which a suppression of at least 15% could be achieved.

### Sorting and functional testing of the four different Treg cell subsets

Venous blood samples (100 ml) from six different healthy, non-transplanted volunteers were collected into EDTA-containing tubes. The whole peripheral blood mononuclear cells (PBMCs) were isolated by Ficoll-Hypaque (Amersham Bioscience) gradient centrifugation. For fluorescence activated cell sorting of the four different Treg cell subsets, CD4^+^CD127^low+/−^CD25^+^-Treg cells were purified using the CD4^+^CD127^low+/−^CD25^+^-Regulatory T cell Isolation Kit II (Miltenyi Biotec) as described above. Respectively, 5×10^5^ cells of the isolated CD4^+^CD127^low+/−^CD25^+^-Treg cells were stained with 15 µl PE-Cy7-conjugated anti-HLA-DR (BD Bioscience) and 50 µl FITC-conjugated anti-CD45RA mouse monoclonal antibodies. Dead cells were excluded, while the remaining CD4^+^CD127^low+/−^CD25^+^-Treg cells were sorted using a FACS-VantageSE-Sorter (BD Bioscience). Thereby, the CD4^+^CD127^low+/−^CD25^+^-Treg cell population was divided into four Treg subsets: DR^high+^CD45RA^−^-, DR^low+^CD45RA^−^-, DR^−^CD45RA^−^-, and naïve DR^−^CD45RA^+^-Treg cells. Subsequently, the suppressive activity of each Treg population was analyzed using the above described suppression assay.

### Statistical analysis

Statistical comparison of the percentages of CD4^+^CD127^low+/−^FoxP3^+^-Treg cells of CD4^+^-T cells, of the percentages of the different Treg subsets (DR^high+^CD45RA^−^-Tregs, DR^low+^CD45RA^−^-Tregs, DR^−^CD45RA^−^-Tregs and naïve DR^−^CD45RA^+^-Tregs) within the total CD4^+^CD127^low+/−^FoxP3^+^-Treg cell pool and of the HLA-DR MFIs between the different patient populations was done using the non-parametric H test of Kruskal and Wallis, which is used for simultaneous comparison of more than two sample populations. Each H test was followed by a Dunn test. Comparison of the suppressive activity of the purified CD4^+^CD127^low+/−^FoxP3^+^-Tregs cells was also done using the Kruskal-Wallis-Test. P<0.05 was considered significant. Statistical analyses and graphs were performed using GraphPad Prism version 5 (San Diego, CA, USA) and BiAS 9.14 for windows (Frankfurt, Germany).
